# Environmental Enrichment Improves Vestibular Oculomotor Learning in Mice

**DOI:** 10.3389/fnbeh.2021.676416

**Published:** 2021-06-15

**Authors:** Jos N. van der Geest, Marcella Spoor, Maarten A. Frens

**Affiliations:** Department of Neuroscience, Erasmus MC, Rotterdam, Netherlands

**Keywords:** environment, motor learning, visual stimulation, VOR, OKR, cerebellum

## Abstract

We assessed the behavioral effects of environmental enrichment on contrast sensitivity, reflexive eye movements and on oculomotor learning in mice that were housed in an enriched environment for a period of 3 weeks. Research has shown that a larger cage and a more complex environment have positive effects on the welfare of laboratory mice and other animals held in captivity. It has also been shown that environmental enrichment affects various behavior and neuroanatomical and molecular characteristics. We found a clear effect on oculomotor learning. Animals that were housed in an enriched environment learned significantly faster than controls that were housed under standard conditions. In line with existing literature, the enriched group also outperformed the controls in behavioral tests for explorative behavior. Meanwhile, both visual and reflexive oculomotor performance in response to visual and vestibular stimuli was unaffected. This points toward an underlying mechanism that is specific for motor learning, rather than overall motor performance.

## Introduction

Behavioral, cellular and molecular studies have revealed significant effects of environmental enrichment on rodents. Rearing these animals in larger, more complex environments results in changes in brain structure and function, including increased brain weight, dendritic branching, neurogenesis, gene expression, and improved learning and memory (Lewis, 2004; Fan et al., 2016; Bhagya et al., 2017; Sager et al., 2018).

The classic definition of environmental enrichment is “a combination of complex inanimate and social stimulation” (Rosenzweig et al., 1978). Leach et al. (2000) suggested that any change to the housing system that increases the frequency and diversity of positive natural behaviors, decreases the occurrence of abnormal behavior, maximizes the utilization of the environment, or increase the animal’s ability to cope with challenges of captivity, qualifies the environment as being enriched. Classic behavioral tests, such as the Open Field Test (Hall, 1934), the Hole Board test (File and Wardill, 1975a), and the Light/Dark test (Crawley and Goodwin, 1980), show that mice show less signs of anxiety and hyperactivity in response to novelty, when housed in an enriched environment (Würbel, 2001). At the behavioral level, enrichment enhances learning and memory (Schrijver et al., 2002), reduces memory decline in aged animals (Bennett et al., 2006), decreases anxiety, and increases exploratory activity (Chapillon et al., 1999; Roy et al., 2001).

Various studies have shown that enrichment increases dendritic branching and length, the number of dendritic spines and the size of synapses on some neuronal populations (Leggio et al., 2005; Sager et al., 2018). Many of these cellular changes are also consistent with enrichment-induced alterations in the expression of genes involved in synaptic function and cellular plasticity (Rampon et al., 2000). Enrichment can increase levels of neurotrophins, such as brain-derived neurotrophic factor (BDNF) and nerve growth factor (NGF), which play integral roles in neuronal signaling (Pham et al., 1999; Ickes et al., 2000; Novkovic et al., 2015; Fan et al., 2016). Furthermore, enrichment results in increased synaptic strength, including specific forms of synaptic plasticity such as long-term potentiation (Foster et al., 1996; Foster and Dumas, 2001; Bhagya et al., 2017; Cortese et al., 2018).

Improvements in motor coordination have been observed in mice, which were housed in an enriched environment, as opposed to mice kept in regular housing system (Chapillon et al., 1999). An enriched environment causes specific alterations in the biophysical properties of cerebellar granule cells, allowing for higher firing frequencies. These alterations were accompanied by superior motor skills (Hallermann et al., 2019). The volume of the cerebellum, along with other brain areas, increases as a result of environmental enrichment (Scholz et al., 2015).

In the present study, the effects of environmental enrichment on visual and sensorimotor function, and cerebellum dependent learning were measured. We focus on the question whether the standard barren housing environment in labs that do behavioral research might affect (oculomotor) behavior that is thought to represent the functional capabilities of mice in general. Mice, being afoveate mammals, show robust cerebellum dependent gaze-stabilizing eye movements like the optokinetic reflex (OKR), which prevents the image of the surroundings to slip across the retina during movement of the visual scene and the vestibulo-ocular reflex (VOR), which responds to vestibular stimulation (Collewijn, 1981). We use the OKR response to measure the animal’s visuo-motor functioning. The VOR is induced to analyze the functioning of the vestibular system. We studied oculomotor learning by creating a mismatch between visual and vestibular stimulation. In addition, we assessed visual acuity and explorative behavior to control for possible mediating effects of enrichment on oculomotor performance.

We hypothesized that mice housed in an enriched environment showed normal visual, visuo-motor and vestibular function, but improved cerebellar-dependent motor learning.

## Materials and Methods

### Animals

In total eighteen C57/Bl6 mice (Charles River Netherlands) were used in the present study. All mice were male, which is common practice in studies on mice behavior. Two age cohorts of mice were measured, aged 10 weeks (*n* = 6), and aged 16 weeks (*n* = 12). The data were pooled, however, as there were no significant differences between these age cohorts. Experiments were conducted with approval of the local ethics committee and in accordance with the European Communities Council Directive (2010/63/EU).

#### Housing Conditions

**FIGURE 1:**
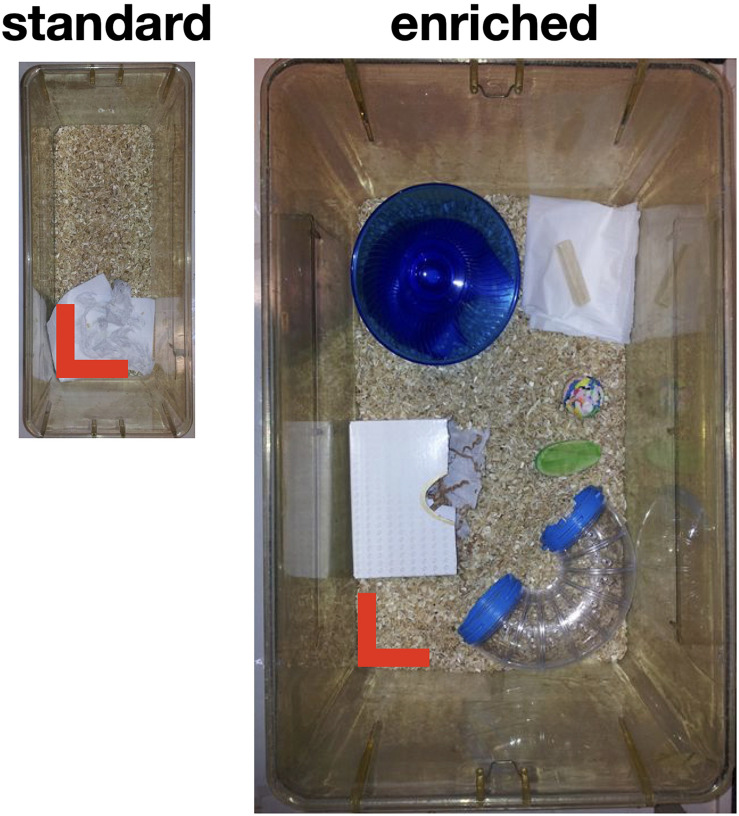
Housing of animals. Standard housing cages, being 33 × 15 cm^2^ in size (13 cm height), compared to our Enriched housing cages of 48 × 38 cm^2^ in size (21 cm height). Note that the pictures are roughly in the same scale. The red bars indicate 10 cm.

Littermates were randomly assigned to one of the two experimental groups (standard vs. enriched housing), containing nine animals each (Figure 1). The mice kept in standard housing conditions were housed with three littermates in a Makrolon 1L cage (33 × 15 × 13 cm), containing one tissue for nest building. The enriched mice were housed with three littermates in a larger Makrolon type IV cage (48 × 38 × 21 cm). Besides sawdust, this cage contained wood shavings, a running wheel, a shelter, plastic tunnel and colored wooden blocks. Throughout the enrichment period, the objects were replaced once a week.

Mice were housed in their respective environments for about 5 weeks, the total duration of the experimental protocol. All mice received the same type of food and water, which were provided *ad libitum*, and were housed on a 12-h light/12-h dark cycle until the end of the protocol.

#### Surgical Preparation

After 3 weeks of housing, the mouse was prepared for the eye movement tests by attaching, under anesthesia, a pedestal to their skull using a construct made of a micro glass composite. This pedestal was necessary for head fixation during the experiments. The full procedure is described in van Alphen et al. (2009). After the surgery, mice were placed back in their own cage (barren or enriched) and had a week to recover.

### Experimental Procedures

In each mouse we assessed explorative behavior, visual functioning, and compensatory oculomotor performance and learning.

#### Explorative Behavior

We subjected every mouse to three tests of explorative behavior, whose outcomes have been well established to be affected by environmental enrichment (Würbel, 2001; Wolfer et al., 2004). In all these tests, mice were released in the corner of an environment of 72 × 72 × 40 cm in size and filmed for 10 min. The recorded videos were scored by hand.

In the Open Field Test (Hall, 1934, 1936), the mice were released in the corner of the lighted environment. We scored how often the mice entered the central area of 18 × 18 cm. In the Hole Board test (File and Wardill, 1975b,a), the bottom of this lighted environment had a rectangular grid of 6-by-6 holes (2.5 cm in diameter) at regular distances. We scored the number of times that a mouse explored a hole by putting its head in it. In the Light/Dark test (Crawley and Goodwin, 1980; Crawley, 2007), half of the environment was dark, and the other half was lighted. There was one small opening between both compartments that was blocked at the onset of the experiments. A mouse was released in the dark compartment and after 5 min of acclimatization the small opening was unblocked. We recorded the total amount of time spent in the light compartment, in the 10 min after that.

### Stimulus and Recording Setup

To assess visual functioning, compensatory oculomotor performance, and oculomotor learning, we recorded eye movements during vestibular and/or optokinetic stimulation.

#### Visual Stimulation

Visual stimulation was created using a modified Electrohome Marquee 9000 CRT projector (Christie Digital Systems, Cypress, CA, United States), which projected stimuli via mirrors onto three transparent anthracite-colored screens (156^∗^125 cm) that were placed in a triangular formation around the recording setup [see van Alphen et al. (2009), for more details]. This created a green monochrome panoramic display, fully surrounding the animal.

#### Vestibular Stimulation

Vestibular stimulation was provided via a motorized turntable Mavilor-DC motor 80 (Mavilor Motors S.A., Barcelona, Spain) on which the mouse and eye movement recording system were mounted. The driving signal of both the visual and vestibular stimulation, which specified the required position, was computed and delivered by a CED Power1401 data acquisition interface (Cambridge Electronic Design, Cambridge, United Kingdom) with a resolution of 0.1° and 0.01 s.

#### Eye Movement Recording

Position of the left eye was recorded with an infrared video system (Iscan ETL-200). Images of the eye were captured at 120 Hz with an infrared sensitive CCD camera [see van Alphen et al. (2009) for more details]. Mice were immobilized by placing them in a plastic tube, with the head pedestal bolted to a restrainer that allowed placing the eye of the mouse in the center of the visual stimulus, in front of the eye position recording apparatus. This mouse holder was mounted on the vestibular table.

Offline data analysis was done in Matlab (version R2019a, the MathWorks, Natick, MA, United States). Recorded eye positions were transformed into a velocity signal. Fast phases and saccades were removed using a velocity threshold of twice the stimulus velocity. For a particular stimulation, we divided eye velocity by stimulus velocity to obtain a gain. An eye movement that perfectly follows a stimulus has a gain of 1 (Collewijn, 1981).

#### Visual Functioning – Contrast Sensitivity

The method used in this study infers contrast sensitivity by measuring how the magnitude (gain) of the OKR varies with different combinations of contrast and spatial frequency. The methods have been described fully in van Alphen et al. (2009). In short, contrast sensitivity was tested by recording eye movements evoked by moving vertically oriented visual sine gratings. A stimulus was made up of a combination of one of seven spatial frequencies (0.03, 0.05, 0.08, 0.17, 0.25, 0.33, or 0.42 cycles per degree) and one of six contrast values (100, 75, 50, 25, 10, or 1%). It was first projected and kept stationary for 1 min, allowing the animal to adjust to changes in the stimulus. Subsequently, the stimulus started to move with a constant velocity of 1.5°/s. After moving to one direction for 2 s, it changed direction and moved in the opposite direction for 2 s. This was repeated six times, yielding 11 changes in direction. The 42 stimulus combinations were presented in random order.

In analyzing the responses, the first 200 ms after stimulus onset and after each change in direction were discarded. Average absolute eye velocity could be divided directly by the (constant) stimulus velocity to calculate a gain value for each stimulus combination.

### Compensatory Oculomotor Performance and Learning

The visual stimulus to induce an optokinetic reflex (OKR) consisted of 1,592 green dots that were equally spaced on a virtual sphere that has its center at the left eye of the mouse. The stimulus oscillated sinusoidally about the earth vertical axis with a constant amplitude of 5° at maximum contrast. By using different oscillation frequencies (0.1, 0.2, 0.4, 0.8, 1.6 Hz) the peak velocity of the stimulus was varied.

The vestibular stimulus to induce a vestibular ocular reflex (VOR) was created by oscillating the mouse about the earth vertical axis at three different frequencies (0.2, 0.6, and 1 Hz) with a constant amplitude of 5° on the turntable.

To induce motor learning, a VOR gain down training paradigm was used, where the visual and vestibular stimuli were presented together and moved at the same speed and exactly in phase at a frequency of 1 Hz and an amplitude of 5°. These training stimuli were presented in six blocks of 20 min. To probe the effects of training, VOR in the dark was measured before the training and after each training block. These test stimuli had the same amplitude and frequency as the training stimuli and were presented for 2 min each.

For OKR, VOR, and VOR learning, the data was analyzed per frequency, by fitting a sinusoid with the frequency of the stimulus through the eye velocity data. Gains were determined by dividing fitted amplitudes by stimulus peak velocities.

### Experimental Protocol

The procedure and order of experiments was the same for all mice. After 3 weeks of housing, the three behavioral tests (Open Field Test, Hole Board, Light/Dark test) were performed on the day before the surgery. A week after surgery, the four other experiments were performed on two measurements days, separated by at least 1 day of rest. On the day before the first measurement day, the mouse was acclimatized to the experimental setup for 15–30 min: they were placed in the setup, but no experiments were performed. On the first day, the OKR and contrast sensitivity were examined. On the second day, the VOR and VOR Learning were examined.

### Statistical Analysis

Group differences in the behavioral tests were statistically assessed using one-sided non-parametric tests (Mann-Whitney). Group differences in the compensatory eye movement tests were assessed using repeated measures ANOVA’s with one between-subject factor Group (2 levels: standard vs. enriched) and either one within-subject factor Frequency (5 levels for OKR, and 3 levels for VOR, being the stimulus frequencies), or one within-subject factor Block for the VOR Learning test (6 levels). Statistics were performed using JASP (v1.0) and statistical thresholds were set at an alpha level of 5%.

## Results

We compared explorative behavior, compensatory eye movements (OKR and VOR), and VOR Learning between mice housed in an enriched environment (*n* = 9) and mice housed in a standard environment (*n* = 9).

### Explorative Behavior

**FIGURE 2:**
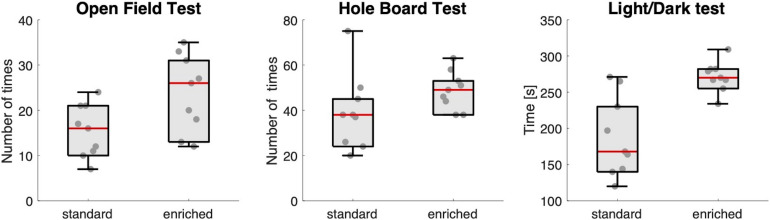
Explorative behavior. Box-and-whisker plots showing the results of the three behavioral tests for the two groups of mice (standard and enriched). Each dot is an individual mouse. The red line in the box is the median value of the group.

Housing conditions did indeed affect explorative behavior: mice in the enriched group showed more explorative and less anxious behavior than mice in the standard housing group (Figure 2). They visited the central part of the floor of the arena in the Open Field Test more often (median ± IQR: 26 ± 17 vs. 16 ± 11, *U* = 16.5, *p* = 0.019), dipped their head more often into one of the holes in the Hole Board test (49 ± 15 vs. 38 ± 23, *U* = 19, *p* = 0.031), and spent more time in the light chamber of the Light/Dark test (270 ± 21 vs. 168 ± 106, *U* = 7, *p* = 0.002).

### Visual Functioning – Contrast Sensitivity

**FIGURE 3:**
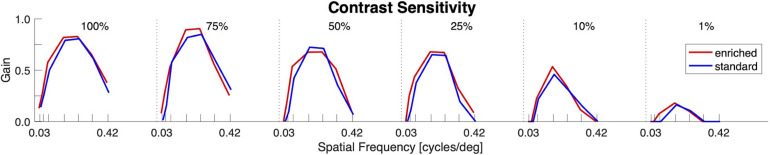
Visual functioning (Contrast Sensitivity). OKR gain as function of spatial frequency for different levels of contrast. No differences between the enriched (red) and standard (blue) housed groups could be observed.

No significant differences in visual functioning between the two groups were observed as reflected by similar optokinetic responses to moving gratings with combinations of different contrasts and spatial frequencies (Figure 3).

#### Compensatory Oculomotor Performance and Learning

**FIGURE 4:**
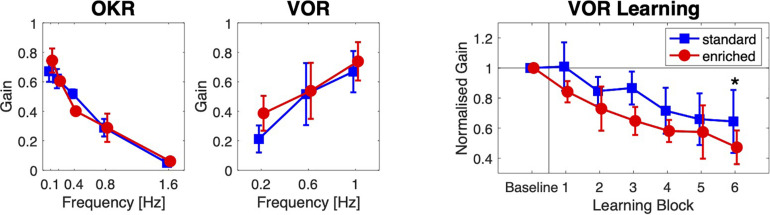
Oculomotor learning. Optokinetic (OKR) and vestibular (VOR) compensatory eye movements, and VOR Learning for the two groups of mice. The OKR and VOR gain are plotted against the stimulation frequency. The mean normalized VOR gain is plotted against Learning Block. Error bars indicate standard deviations (* = *post hoc* significance *p* < 0.05).

Housing conditions did not importantly affect optokinetic (OKR) and vestibulo-ocular (VOR) reflexes, but it did affect VOR Learning (Figure 4).

As expected, the main effect of stimulus frequency was significant for both types of compensatory eye movements: with increasing stimulus frequencies, OKR gain decreased [*F*(4,64) = 722.8, *p* < 0.001, ω^2^ = 0.95], and VOR gain increased [*F*(2,32) = 114.0, *p* < 0.001, ω^2^ = 0.55]. The overall differences in gains between the two groups were not significant [OKR: *F*(1,16) = 0.28, *p* = 0.60, ω^2^ = 0.00; VOR: *F*(1,16) = 1.88, *p* = 0.189, ω^2^ = 0.05]. For both types of eye movements, the interactions between group and stimulus frequency were significant but small [OKR: *F*(4,64) = 12.7, *p* < 0.001, ω^2^ = 0.24; VOR: *F*(2,32) = 4.20, *p* = 0.024, ω^2^ = 0.034].

Vestibulo-ocular reflex gains decreased during the adaptation blocks [effect of block on VOR gain: *F*(5,80) = 26.0, *p* < 0.001, ω^2^ = 0.45] in both groups, but the overall reduction in VOR gain was larger in the enriched group than in the standard housing group [*F*(1,16) = 11.6, *p* = 0.004, ω^2^ = 0.37]. The interaction between Group and Block was not significant [*F*(5,80) = 0.80, *p* = 0.56, ω^2^ = 0.00]. A *post hoc* non-parametric analysis showed that the normalized VOR gain in the last block was smaller in the enriched group (mean ± SD: 0.47 ± 0.11) than in the standard housing group (0.65 ± 0.21, *t* = 2.18, *p* = 0.02).

## Discussion

Environmental improvement has been a topic in rodent research for more than 60 years, inspired by Donald O. Hebb’s finding that the rats he kept as pets, exposed to a more complex environment, showed better performance in learning and memory than his research animals (Brown, 2006). To this we add our finding that mice that are housed in an enriched environment not only show the often-observed surge in explorative behavior, but also seem to show improved oculomotor learning. This enhanced cerebellar-dependent learning was not induced by altered compensatory oculomotor performance or visual functioning of these mice.

Adding structuring to an enlarged cage provided the mice with shelter and increased opportunities for exploration and locomotion. From our results of the standard behavioral tests Open Field Test (OFT), Hole Board (HB), and Light Dark (LD) we can infer that such altered housing conditions indeed did have an effect on measured behavior. We therefore conclude that our experimental manipulations of housing are in good accordance with the existing literature (Würbel, 2001), and therefore consider our experimental manipulation of housing conditions valid.

We found that improved housing conditions did not improve the contrast sensitivity, which is in line with earlier findings of Greifzu et al. (2016). However, it has been previously shown that environmental enrichment from birth does enhance visual acuity in mice (Prusky et al., 2000; Cancedda et al., 2004; Sale et al., 2004). Thus, different environmental conditions could act as indirect mediator for the earliest effects on visual system development. Our study deviates from these findings in that the mice were older (10–16 weeks).

We also observed that an enriched environment did not alter the OKR or the VOR gain. Apparently, these reflexes are sufficiently robust in the C57Bl/6J mice that they do not vary with varying housing conditions. Earlier literature has reported on the effects of enrichment on motor behavior, but this was either about locomotion in disease models (Jadavji et al., 2006; Knieling et al., 2009), or about higher level movement parameters, such as the amount of stereotypical behavior (Powell et al., 2000; Marashi et al., 2003; Turner and Lewis, 2003). Our data seem to be the first to report that the compensatory eye movements are not affected, despite the fact that the animals can move around more in their cages, and therefore presumably also make more head movements.

Mice from the enriched group showed a more enhanced VOR decrease than the mice from the standard housing condition group. Because both the OKR and VOR are unaffected, it is likely that these differences are due to the learning mechanism itself. This VOR gain decrease test is a frequently used test in neuroscience to assess cerebellar learning (Schonewille et al., 2011; Gutierrez-Castellanos et al., 2013; Shin et al., 2014; Das et al., 2017; Inagaki and Hirata, 2017; Voges et al., 2017; Galliano et al., 2018). The results obtained in this study tell us to carefully interpret such data, especially because differences in brain anatomy and function due to housing condition instead of inherent to the animal model tested can be accounted for the obtained differences on behavioral level. Inspection of the data presented in the literature shows that the differences that we find between the standard and enhanced housed groups are in the same order of magnitude as the effects that are often reported in mouse mutants as significant learning deficits.

The present study shows that mice housed in standard environments across all labs that do behavioral research are probably a poor representation of what mice are actually capable of with repect to oculomotor learning. It would be good to know whether the improvement in VOR gain was due to sensory enrichment or voluntary exercise, and also to further dissect which of the changes in the housing cage leads to altered motor learning: if it is the size, the amount of nesting material and/or the additional objects added to the cage. A future study could, for instance, delineate sensory from motor enrichment, by testing the effects of daily exercises outside a standard cage, as it is clear that locomotion modulates cerebellar-dependent learning (Albergaria et al., 2018).

In summary, we observed an effect of enrichment on an often used cerebellar-dependent learning task. We can only speculate on the underlying mechanism that causes the differences in learning speed between the two groups. Experiments have shown that exposure to an enriched environment has been shown to induce robust neuronal plastic changes in both the cerebral as well as in the cerebellar cortex (Angelucci et al., 2009).

Previous studies showed that different housing conditions can cause differences in brain anatomy and function. Enriched rodents have been observed to have increased brain weight and size (Bennett and Rosenzweig, 1969). Also various studies have shown that enrichment increases dendritic branching and length, the number of dendritic spines and the size of synapses on some neuronal populations in the hippocampus and cerebral cortex (Greenough and Volkmar, 1973; Greenough et al., 1973; Faherty et al., 2003; Leggio et al., 2005; Sager et al., 2018). Brain-derived neurotrophic factor (BDNF) levels, which plays a role in cerebellar plasticity, increase under environmental enrichment (Vazquez-Sanroman et al., 2013). Electrophysiological data measured in the hippocampus has shown that differential experience results in increased synaptic strength, including specific forms of synaptic plasticity such as long-term potentiation (LTP; Foster et al., 1996; Foster and Dumas, 2001; Artola et al., 2006) and long- term depression (LTD; Artola et al., 2006). Morphological and electrophysiological data cerebellar data from rodents kept in complex housing conditions confirm these observations (Hallermann et al., 2019).

We conclude that a more complex environment increases exploratory behavior, and improves learning measured by an often-used paradigm in neuroscientific studies to assess learning. These results provide food for thought regarding the potential effects of the conventional housing laboratory animals are usually kept in. Despite it is basic biology that the phenotype of an animal is the product of a complex and dynamic interplay between nature (genotype) and nurture (environment), the latter, a sterile small box, is far less considered into the interpretation of animal experiments.

Standardization is an important concept in neuroscientific research and environmental standardization has become a sort of dogma. It is explicitly encouraged, because between-experiment standardization is thought to increase reproducibility of results, whereas within experiment standardization is thought to increase test sensitivity (van de Weerd et al., 1994). This was also the reason why we used only male mice, because this is the most standard in the mice behavioral literature. Ironically, despite rigorous standardization poor between-laboratory replicability has been revealed (standardization fallacy), which can be causing conflicting findings published (Würbel, 2000).

One could argue that the introduction of a more complex environment may change certain characteristics of animals, and as a consequence, experimental results may not be comparable with previously found results. This, however, should not hamper the introduction of enrichment, as it may be questioned whether the maintenance of animals in unresponsive environments makes them adequate models for extrapolating results (Markowitz and Gavassi, 1995). A growing body of evidence indicates that current approaches to behavioral phenotyping might often produce results that are idiosyncratic to the study in which they were obtained, because the interactive nature of genotype-environment relationships underlying behavioral phenotypes was not taken into account (Würbel, 2001; Olsson and Dahlborn, 2002; Simpson and Kelly, 2011).

Because the effect of a mutation on behavior might substantially differ depending on the animals’ genetic or environmental background, and because idiosyncratic results are scientifically useless, it is absolutely crucial that variation of experimental conditions forms an integral part of behavioral phenotyping research (Würbel, 2000). Our study shows that experimental background conditions that may seem unrelated to the scientific questions at hand could importantly affect the interpretation of obtained results.

## Data Availability Statement

The raw data supporting the conclusions of this article will be made available by the authors, without undue reservation.

## Ethics Statement

The animal study was reviewed and approved by the Erasmus MC Animal Ethics Committee.

## Author Contributions

MS and MF designed the study. MS collected the data. MS and JG analyzed the data and drafted the manuscript. JG and MF finalized the manuscript. All authors contributed to the article and approved the submitted version.

## Conflict of Interest

The authors declare that the research was conducted in the absence of any commercial or financial relationships that could be construed as a potential conflict of interest.
